# Rotator Cuff Repair: Racial Disparities in Operative Time and Utilization of Arthroscopic Techniques

**DOI:** 10.7759/cureus.65673

**Published:** 2024-07-29

**Authors:** Steven Liu, Allen Bramian, Rachel A Loyst, Kenny Ling, Christian Leonardo, David Komatsu, Edward D Wang

**Affiliations:** 1 Department of Orthopedic Surgery, Stony Brook University, Stony Brook, USA; 2 Department of Orthopedic Surgery, Stony Brook Hospital, Stony Brook, USA

**Keywords:** complications’, operative time, arthroscopic shoulder surgery, racial disparities, rotator cuff repair surgery

## Abstract

Background

Racial disparities are prevalent within the field of orthopedics and include the utilization of varying resources as well as outcomes following surgery. This study investigates racial differences between Black and White patients in the surgical treatment of rotator cuff repair (RCR) and 30-day postoperative complications following RCR.

Materials and methods

Data were drawn from the American College of Surgeons National Surgical Quality Improvement Program (ACS-NSQIP) database to create a study population consisting of Black and White patients who underwent RCR between 2015 and 2019. A bivariate analysis was conducted to compare patient demographics and comorbidities. Multivariate logistic regression, controlling for all significantly linked patient demographics and comorbidities, was performed to examine the relationships between Black race and complications.

Results

Our analysis included 32,073 patients, of whom 3,318 (10.3%) were Black and 28,755 (89.7%) were White. The female gender, younger age groups, greater BMI groups, ASA classification ≥3, cigarette use, and comorbid congestive heart failure (CHF), diabetes, and hypertension were all significantly associated with patients who identified as Black. We found no significant differences in 30-day postoperative complications between Black and White patients. Furthermore, Black patients were found to be independently associated with a greater likelihood of undergoing arthroscopic RCR versus open RCR, as well as experiencing a longer total operation time of ≥80 minutes.

Conclusions

We report no differences in 30-day postoperative complications between Black and White patients undergoing RCR between 2015 and 2019. However, Black race was independently associated with higher rates of arthroscopic RCR and longer operative times.

## Introduction

Rotator cuff tears are a common cause of shoulder pain and dysfunction, with treatment including both nonoperative management and surgical repair. Rotator cuff repair (RCR) is a surgical procedure commonly indicated for patients with acute full-thickness tears, significant functional disability, or failure of nonsurgical therapy [1-3]. Surgical techniques used in RCR include arthroscopy, open surgery, and mini-open surgery, of which arthroscopic RCR is the most common [4-8]. The yearly volume of RCR procedures continues to rise in the United States, with a dramatic increase from approximately 41 per 100,000 in 1996 to approximately 98 per 100,000 in 2006 [9]. An analysis of RCR in a cohort of patients 18-64 years of age with at least one year of commercial insurance coverage reported that the proportion of arthroscopic RCR grew from 60% to 83% of all RCRs between 2007 and 2016, showing that arthroscopy has become the preferred operative modality to treat RCR [8].

Recent analyses have revealed the influence of racial disparities on orthopedic care, including lower utilization rates of total shoulder arthroplasty (TSA), total hip arthroplasty (THA), and total knee arthroplasty (TKA) in Black patient populations [10-18]. Studies have revealed that, relative to White patients, Black patients are more likely to experience postoperative complications following total joint arthroplasty (TJA) [19,20]. A recent study investigating the utilization of RCR for rotator cuff pathology in New York State found that the Black race was independently associated with lower rates of RCR following diagnosis of a rotator cuff tear [21]. While studies investigating differences in postoperative outcomes following RCR between White and Black populations are limited, they have shown that Black patients are more likely to experience longer operative times and increased hospital length of stay following RCR [15].

Given the lower utilization rates of RCR in Black patient populations and limited evidence comparing postoperative outcomes between Black and White patients following RCR, this investigation was conducted to explore the relationship between race and complications following RCR as its prevalence increases. This study aimed to compare early postoperative complications following RCR between White and Black patients. We hypothesized that Black patients experience a greater rate of complications following RCR as compared to White patients.

## Materials and methods

We used the American College of Surgeons National Surgical Quality Improvement Program (ACS-NSQIP) database from the years 2015 to 2019 to search for patients who underwent RCR. Since the NSQIP database is fully de-identified, our study did not require approval from the university’s Institutional Review Board. The NSQIP database contains data from over 600 hospitals nationwide, with data procurement completed by trained surgical clinical reviewers and regular audits to ensure accuracy and reliability.

**Figure 1:**
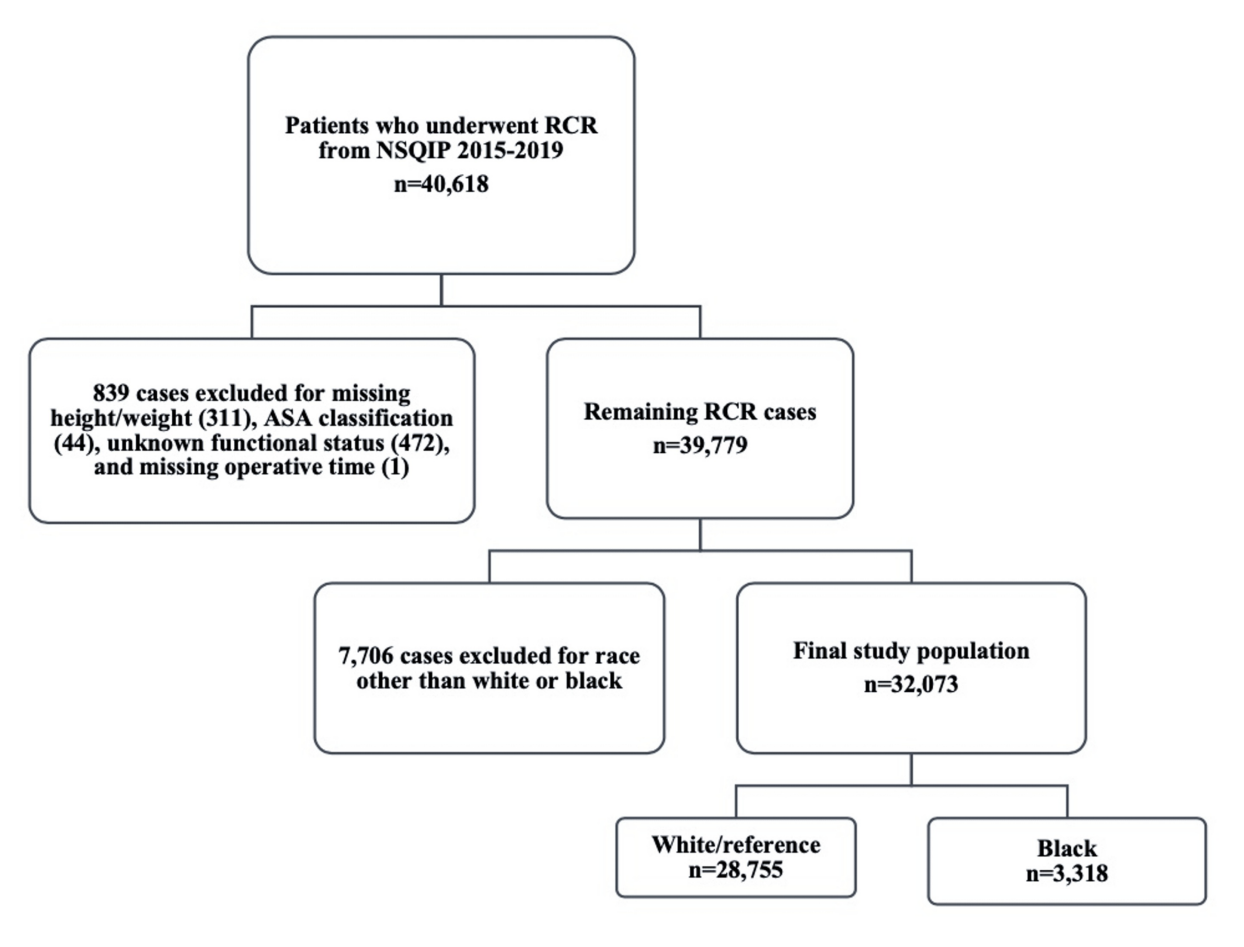
Case selection schematic. RCR: rotator cuff repair; NSQIP: National Surgical Quality Improvement Program; ASA: American Society of Anesthesiologists.

The current procedural terminology codes for open RCR (23412, 23410) and arthroscopic RCR (29827) were used to identify 40,618 patients who underwent RCR between 2015 and 2019. Data from the years 2020 to 2021 were not included in the study to exclude the effects of the COVID-19 pandemic. The exclusion criteria inherent to the NSQIP database exclude all cases of patients younger than 18 years of age. Eight hundred and thirty-nine cases were excluded for missing height or weight, American Society of Anesthesiologists (ASA) classification, functional status, or operative time. Seven thousand seven hundred and six cases were excluded for a patient race other than White or Black (Figure 1). The remaining study population of 32,073 was divided into White and Black races.

Variables collected in this study included patient demographics (gender, age, BMI, functional status, ASA classification, smoking status, and preoperative steroid use), comorbidities (congestive heart failure [CHF], diabetes, hypertension, severe chronic obstructive pulmonary disease [COPD], bleeding disorders, and disseminated cancer), and surgical characteristics (operative procedure such as open or arthroscopic RCR and total operative time). Thirty-day postoperative complication data were also collected for the following complications: sepsis, septic shock, pneumonia, unplanned reintubation, urinary tract infection (UTI), cardiac arrest, myocardial infarction (MI), stroke, blood transfusions, deep vein thrombosis (DVT), pulmonary embolism (PE), time spent on ventilator >48 hours, surgical space infection (SSI), wound dehiscence, acute renal failure, *Clostridioides difficile* infection, non-home discharge, readmission, unplanned reoperation, length of stay >2 days, and mortality.

SPSS Software version 26.0 (IBM Corp., Armonk, NY, USA) was used to conduct statistical analyses on the collected data. Bivariate logistic regression compared differences in demographics, comorbidities, and complication rates between racial cohorts. Multivariate logistic regression identified independently significant associations between complications between cohorts on bivariate analysis. This process was performed by: (1) all significant demographic and comorbidity variables by racial cohort were initially included; (2) multivariate analysis was repeatedly conducted, and with each iteration, one variable with the largest p-value was eliminated; and (3) this process was continued until all variables remaining were statistically significant. Odds ratios (OR) were reported with 95% confidence intervals (CI), and p < 0.05 (alpha of 0.05) was used as the level of statistical significance.

All statistical analyses were conducted using SPSS Software version 26.0 (IBM Corp., Armonk, NY, USA). Patient demographics and comorbidities were compared between cohorts using bivariate logistic regression. Multivariate logistic regression was conducted to identify significant associations between patient variables and both operative procedure and total operative time in the Black patient population. The analysis proceeded in a stepwise manner: (1) patient demographics and comorbidities significantly linked to Black race were initially included; (2) variables were sequentially removed, starting with the highest p-value, until only statistically significant associations remained. ORs with 95% CIs were reported, and statistical significance was defined as p < 0.05 (alpha of 0.05).

## Results

Black patients were significantly associated with female gender (p < 0.001), younger age groups (p < 0.001), greater BMI groups (p < 0.001), ASA classification ≥ 3 (p < 0.001), smokers (p = 0.010), comorbid CHF (p < 0.001), diabetes (p < 0.001), and hypertension (p < 0.001) compared to White patients (Table 1). White patients were significantly associated with bleeding disorders compared to Black patients (p = 0.034). Patients who identified as Black were significantly more likely to undergo arthroscopic RCR versus open RCR (p = 0.002) and to experience total operation times lasting 80 minutes or longer (p < 0.001).

**Table 1:** Demographic characteristics and pre-existing medical conditions among Black patients compared to White patients. The P-values that are bolded indicate statistical significance with p < 0.05.

	White	Black	p-value
	Number (%)	Number (%)	
Overall	28,755 (100.0)	3,318 (100.0)	
Sex			
Female	11,801 (41.0)	1,731 (52.2)	<0.001
Male	16,954 (59.0)	1,587 (47.8)	
Age			
18–39	1,225 (4.3)	224 (6.8)	<0.001
40–64	18,167 (63.2)	2,484 (74.9)	
65–79	7,526 (26.2)	524 (15.8)	
≥80	1837 (6.4)	86 (2.6)	
BMI (kg/m^2)			
<18.5	111 (0.4)	10 (0.3)	<0.001
18.5–29.9	14,143 (49.2)	1,260 (38.0)	
30–34.9	7,930 (27.6)	983 (29.6)	
35–39.9	3,871 (13.5)	607 (18.3)	
≥40	2,700 (9.4)	458 (13.8)	
Functional status prior to surgery			
Dependent	120 (0.4)	18 (0.5)	0.298
Independent	28,635 (99.6)	3,300 (99.5)	
ASA classification			
≤2	18,217 (63.4)	1,970 (59.4)	<0.001
≥3	10,538 (36.6)	1,348 (40.6)	
Smoker			
No	24,442 (85)	2,764 (83.3)	0.010
Yes	4,313 (15)	554 (16.7)	
Steroid use			
No	28,132 (97.8)	3,234 (97.5)	0.176
Yes	623 (2.2)	84 (2.5)	
Comorbidities			
CHF	30 (0.1)	13 (0.4)	<0.001
Diabetes	4,719 (16.4)	802 (24.2)	<0.001
Hypertension	13,384 (46.5)	1,999 (60.2)	<0.001
COPD	998 (3.5)	102 (3.1)	0.235
Bleeding disorder	449 (1.6)	36 (1.1)	0.034
Disseminated cancer	16 (0.1)	2 (0.1)	0.915
Operative procedure			
Open	4,529 (15.8)	453 (13.7)	0.002
Arthroscopic	24,226 (84.2)	2,865 (86.3)	
Total operation time (minutes)			
0–79	15,217 (52.9)	1,490 (44.9)	<0.001
80–128	9,371 (32.6)	1,225 (36.9)	
≥129	4,167 (14.5)	603 (18.2)	

Patients who identified as Black were not found to have significantly different rates of 30-day postoperative complications (Table 2). Furthermore, these patients were independently associated with a greater likelihood of undergoing arthroscopic RCR (OR 1.15, 95% CI: 1.03-1.28; p = 0.010) versus open RCR and experiencing a longer total operation time of ≥ 80 minutes (OR 1.26, 95% CI: 1.20-1.32; p < 0.001) (Table 3).

**Table 2:** Significant predictors of arthroscopic rotator cuff repair in patients of Black race.

Characteristic	OR, p-value (95% CI)
Sex	
Female	Reference
Male	1.24, 0.039 (1.01–1.52)
Smoker	
No	Reference
Yes	1.62, <0.001 (1.27–2.08)
Total operation time (minutes)	
0–79	Reference
≥80	2.06, <0.001 (1.68–2.53)

**Table 3:** Comparison of 30-day postoperative complications between Black and White patients through bivariate analysis. The bolded p-values indicate statistical significance with p < 0.05.

	White	Black	p-value
	Number (%)	Number (%)	
Any complication	866 (3.0)	102 (3.1)	0.842
Sepsis	12 (0.0)	3 (0.1)	0.231
Septic shock	4 (0.0)	0 (0.0)	0.999
Pneumonia	42 (0.1)	2 (0.1)	0.221
Unplanned reintubation	14 (0.0)	2 (0.1)	0.778
UTI	57 (0.2)	5 (0.2)	0.556
Cardiac arrest or MI	25 (0.1)	4 (0.1)	0.544
Stroke	9 (0.0)	1 (0.0)	0.971
Blood transfusions	8 (0.0)	0 (0.0)	0.999
DVT	42 (0.1)	7 (0.2)	0.367
PE	48 (0.2)	8 (0.2)	0.335
On ventilator >48 hours	8 (0.0)	3 (0.1)	0.082
SSI	77 (0.3)	5 (0.2)	0.212
Wound dehiscence	6 (0.0)	1 (0.0)	0.734
Acute renal failure	3 (0.0)	0 (0.0)	0.999
*C. difficile* infection	10 (0.0)	1 (0.0)	0.891
Non-home discharge	0.6 (0.6)	23 (0.7)	0.472
Readmission	325 (1.1)	41 (1.2)	0.588
Unplanned reoperation	107 (0.4)	9 (0.3)	0.361
Length of stay >2 days	247 (0.9)	37 (1.1)	0.137
Mortality	7 (0.0)	1 (0.0)	0.842

Independently significant predictors of a longer total operative time (≥80 minutes) in Black patients were male sex (OR 1.66, 95% CI: 1.44-1.91; p < 0.001), ASA classification ≤ 2 (OR 1.21, 95% CI: 1.05-1.40; p = 0.008), and arthroscopic RCR operative procedure (OR 2.06, 95% CI: 1.68-2.53; p < 0.001) (Table 4). Independently significant predictors of undergoing an arthroscopic RCR procedure in Black patients were male sex (OR 1.24, 95% CI: 1.01-1.52; p = 0.039), smoking status (OR 1.62, 95% CI: 1.27-2.08; p < 0.001), and total operative time ≥ 80 minutes (OR 2.06, 95% CI: 1.68-2.53; p < 0.001) (Table 5).

**Table 4:** Multivariate analysis of 30-day operative factors in patients of Black race compared to patients of White race. Bold p-values indicate statistical significance with p < 0.05.

	Black
	OR, p-value (95% CI)
Arthroscopic RCR operative procedure	1.15, 0.010 (1.03–1.28)
Total operation time ≥80 minutes	1.26, <0.001 (1.20–1.32)

**Table 5:** Significant predictors of total operative time ≥80 minutes for patients of Black race.

Characteristic	OR, p-value (95% CI)
Sex	
Female	Reference
Male	1.66, <0.001 (1.44–1.91)
ASA classification	
≤2	1.21, 0.008 (1.05–1.40)
≥3	Reference
Operative procedure	
Open	Reference
Arthroscopic	2.06, <0.001 (1.68–2.53)

## Discussion

Our retrospective study cohort of 32,073 patients, 10.3% Black and 89.7% White, revealed no significant differences in 30-day postoperative complications after RCR between the Black and White races. However, compared to White patients, Black patients were more likely to undergo an arthroscopic procedure for RCR and were more likely to have a longer operation time. Factors predicting longer operative time in Black patients were male sex, ASA classification ≤ 2, and arthroscopic RCR. Factors predicting arthroscopic RCR were male sex, smoking, and longer operative time.

RCR is an effective treatment for rotator cuff tears and may be performed either arthroscopically or with an open approach, yielding comparable successful outcomes [22-24]. However, arthroscopic repair has quickly become the more frequently used technique [8,9]. Despite the rise in total RCR case volume, studies have revealed lower rates of RCR utilization in the Black population [14,21]. In contrast, a study investigating racial disparities in the utilization of arthroscopic RCR found that the proportion of Black patients undergoing arthroscopic RCR significantly increased from 7.4% in 2010 to 10.4% in 2019 [15].

Historically, pronounced racial disparities have been documented in orthopedic surgical procedures. Specifically, studies have shown that Black patients often require more intensive postoperative rehabilitation in addition to experiencing higher rates of non-home discharge, venous thromboembolism, blood transfusions, readmission, and mortality compared to White patients [16,19,20,25,26]. One study found that the difference in readmission rates between Black and White patients following TJA increased from 6% to 24% from 1991-2008 [16]. Other studies have reported lower utilization rates of TJA in Black patients compared to White patients [10,11,16,17,26,27]. Furthermore, utilization of outpatient TJA has increased from 0.4% to 10.2% in White patients while increasing from 0.6% to only 5.9% in Black patients between 2011 and 2019 [11]. These studies help to illustrate the pattern of racial disparity in orthopedic care between Black and White populations historically.

In the present study, we found that Black race was an independent predictor of undergoing arthroscopic RCR and an independent predictor of longer total operation times. These findings agree with prior studies that revealed an improvement in arthroscopic RCR utilization rates in Black patient populations [15]. In addition, our study found significantly higher rates of female gender, younger age groups, greater BMI groups, ASA classification ≥ 3, smoking, CHF, diabetes mellitus, and hypertension in Black patients who underwent RCR. These findings agree with prior studies that have reported greater rates of comorbidities in Black patient populations undergoing orthopedic procedures [12,15,17,28,29].

Longer RCR operative times observed in Black patients compared to White patients warrant concern, as longer operation times have been shown to increase the risk of postoperative complications [30,31]. Notably, arthroscopic RCR takes longer on average than open repairs [4]. This difference of 89 minutes for arthroscopic repairs compared to 76 minutes for open repairs, in combination with our observation that Black patients were more likely to undergo arthroscopic repair, offers a partial explanation for the longer operative times experienced by Black patients [4]. Other factors leading to longer operative times may include increased case complexity or a high BMI [32].

The higher utilization of arthroscopic RCR versus open RCR in Black patients compared to White patients may also be partly explained by the increased rate of comorbidities, including CHF, diabetes, and hypertension. Since chronic comorbidities are associated with higher rates of postoperative complications, Black patients may be more likely to undergo a less invasive arthroscopic procedure to attenuate the increased risk of chronic medical comorbidities.

Racial disparities persist throughout the field of medicine. Numerous studies have pointed toward lower surgery utilization rates and increased postoperative complications in Black patients receiving orthopedic care as compared to White patients [10,11,15,26]. The mechanisms driving these disparities are complex and have been suggested to arise from a lack of education and cultural competence among providers, differences in patient socioeconomic status, limited access to quality healthcare, and differences in baseline health [17,33]. Nevertheless, our study did not find any statistically significant differences in postoperative complications between Black and White patients following RCR.

This study was limited to the information contained within the NSQIP database. The NSQIP database captures and reports postoperative outcomes within a 30-day period from the time of operation. This limits our analysis to a narrow timeframe and may fail to include adverse events that occurred outside of the 30-day period, including effects of long-term shoulder function and patient follow-up. In addition, selection criteria determined patient race according to the first race listed in the medical record. This method of categorization fails to accurately represent patients of mixed race and may have resulted in heterogeneity within our two study populations that were intended to remain homogeneous. The NSQIP database does not report factors such as the experience of the surgeon, race of the surgeon, institution where the procedure was performed, and postoperative rehabilitation plans. These factors may have provided further insight into the results of our study, especially in the context of the longer operative times observed in the Black patient population.

## Conclusions

Black race was not significantly associated with any differences in 30-day postoperative complications after RCR compared to the White race. However, Black patients were more likely to undergo an arthroscopic procedure for RCR and were more likely to have a longer operation time. Factors predicting longer operative time in Black patients were male sex, ASA classification ≤ 2, and arthroscopic RCR. Factors predicting arthroscopic RCR were male sex, smoking, and longer operative time. As the surgical volume for RCR continues to grow, racial disparities must continue to be investigated to address inequalities and provide equitable care.
